# Editorial: The neuroscience of Parkinson's disease: exploring causes, symptoms, and potential treatments

**DOI:** 10.3389/fnhum.2025.1609635

**Published:** 2025-04-28

**Authors:** Jurgen Germann, Sandra Neumann

**Affiliations:** ^1^Krembil Brain Institute, Toronto, ON, Canada; ^2^Center for Advancing Neurotechnological Innovation to Application (CRANIA), Toronto, ON, Canada; ^3^Institute of Biomedical Engineering, University of Toronto, Toronto, ON, Canada; ^4^Bristol Medical School, University of Bristol, Bristol, United Kingdom

**Keywords:** Parkinson's disease, neurodegeneration, non-motor symptom, therapeutic strategy, neuroimaging, biomarkers

Parkinson's disease (PD) is a chronic and progressive neurodegenerative disorder, best known for its motor symptoms but increasingly recognized for its broad and often debilitating non-motor manifestations (Poewe et al., 2017; Schapira et al., 2017; Blesa et al., 2022). The pathological hallmark of PD is the loss of dopaminergic neurons in the substantia nigra (Braak et al., 2003; Dickson, 2012). PD encompasses a wide range of physiological and psychological symptoms that significantly impact patients and their loved ones (Luo et al., 2024). While the neural mechanisms driving PD remain incompletely understood, research continues to unravel its complex etiology—spanning genetic, environmental, and lifestyle-related factors.

This timely collection brings together new insights from across the neuroscientific spectrum—molecular, structural, behavioral, and technological—aimed at deepening our understanding of PD and advancing clinical care. From emerging biomarkers and endophenotypes to novel therapeutic strategies and real-world monitoring tools, the articles in Research Topic reflect the interdisciplinary spirit of PD research today.

The systematic review and meta-analysis by Zheng et al. examined the clinical impact of REM sleep behavior disorder (RBD) in PD. Analyzing data from over 5,600 patients, the study demonstrates that individuals with RBD experience significantly worse motor and non-motor symptoms, including cognitive impairment, hallucinations, anxiety, and depression. These findings underscore the value of RBD as a potential marker of a more severe PD phenotype, advocating for earlier detection and tailored interventions in this subgroup.

Extending the exploration of cognitive and non-motor features, Pavelka et al. present a cross-sectional analysis from the Luxembourg Parkinson's Study, focusing on individuals with idiopathic PD who experience freezing of gait (FOG). Their data suggest that FOG is associated with a distinct clinical profile, marked by greater motor complications and cognitive impairments, particularly in visuospatial and memory functions. The study introduces a possible non-motor-dominant endophenotype, reinforcing the need to recognize heterogeneity within PD and its implications for disease progression and personalized care.

In a related effort to identify structural neural correlates of motor and non-motor symptoms, Ren et al. employ advanced neuroimaging techniques, including diffusion tensor imaging and graph-theoretical analysis, to assess white matter integrity in PD. Their findings reveal significant disruptions in thalamic-limbic circuits and reduced global and local efficiency in brain networks, which correlate with gait impairment and neuropsychiatric symptoms. The study points to the hippocampus and orbitofrontal cortex as potential therapeutic targets while also supporting the utility of network-level biomarkers for clinical monitoring.

At the molecular level, Lin et al. investigate the neuroprotective potential of synthetic coumarin-chalcone derivatives *in vitro*. Their study highlights two compounds, LM-021 and LM-036, which exhibit potent anti-inflammatory and antioxidant properties by modulating NLRP1 and NLRP3 inflammasomes in microglial and neuronal cell lines. These compounds also promote neuronal viability and neurite outgrowth, suggesting their promise for future therapeutic development aimed at targeting both neuroinflammation and oxidative stress—two key mechanisms implicated in PD pathogenesis.

Adding a complementary systems-level view, Zhao et al. conducted a bibliometric and visual analysis of global research trends on acupuncture for PD. Covering nearly three decades of literature, the study identifies major contributors, emerging keywords, and thematic clusters such as neuroinflammation, non-motor symptoms, and brain-gut peptides. While research output has grown, the authors highlight the need for more rigorous randomized controlled trials and stronger international collaboration to integrate acupuncture into evidence-based PD care.

In the realm of digital health, Rodríguez-Martín and Pérez-López review the current landscape of wearable devices for PD symptom monitoring. The article evaluates several commercial systems—including PKG™, Kinesia™, PDMonitor™, and STAT-ON™—and discusses their capabilities in tracking tremor, dyskinesia, gait disturbances, and ON/OFF fluctuations. Although these tools offer promise for real-world, continuous monitoring, the authors emphasize barriers to adoption, such as lack of standardization, limited validation, and low clinician confidence in device-generated data. The review calls for stronger clinical integration, regulatory clarity, and clinician training to unlock the full potential of wearable technologies.

On the therapeutic front, Ruver-Martins et al. present a case series exploring the effects of low-dose cannabis extract on non-motor symptoms in PD. Their findings suggest that a combination of THC and CBD can improve insomnia and may enhance cognitive performance in some patients, with minimal side effects. Although preliminary, the study opens new avenues for addressing treatment-resistant non-motor symptoms and underscores the importance of further trials to evaluate cannabinoid-based interventions in PD management.

Finally, Vilhalva et al. offer a novel look at the neurophysiological effects of dance-based therapy. Using EEG recordings, they show that participation in structured dance classes enhances Mu rhythm desynchronization—an indicator of mirror neuron system activity—during the observation of both choreographed and everyday movements. These results support the use of dance not only as a motor rehabilitation tool but also as a means to engage cognitive and sensorimotor networks, with potential benefits for motor planning and functional mobility in PD.

Together, the articles present a comprehensive and multi-dimensional view of Parkinson's disease. They highlight critical progress in identifying novel biomarkers, understanding motor and non-motor phenotypes, testing emerging therapies, and leveraging technology for personalized care. This Research Topic reaffirms the need for interdisciplinary approaches to unravel the complexity of PD and drive innovation in both research and clinical practice. We extend our sincere gratitude to all contributing authors and reviewers for their dedication and insight. We hope this Research Topic serves as a valuable resource for scientists, clinicians, and policymakers committed to improving the lives of those affected by Parkinson's disease.

## References

[B1] Blesa J. Foffani G. Dehay B. Bezard E. Obeso J. A. (2022). Motor and non-motor circuit disturbances in early Parkinson disease: which happens first? Nat. Rev. Neurosci. 23, 115–128. 10.1038/s41583-021-00542-9 34907352

[B2] Braak H. Del Tredici K. Rüb U. de Vos R. A. I. Jansen Steur E. N. H. Braak E. (2003). Staging of brain pathology related to sporadic Parkinson's disease. Neurobiol. Aging 24, 197–211. 10.1016/s0197-4580(02)00065-9 12498954

[B3] Dickson D. W. (2012). Parkinson's disease and Parkinsonism: neuropathology. Cold Spring Harb. Perspect. Med. 2, a009258. 10.1101/cshperspect.a009258 22908195 PMC3405828

[B4] Luo Y. Qiao L. Li M. Wen X. Zhang W. Li X. (2024). Global, regional, national epidemiology and trends of Parkinson's disease from 1990 to 2021: findings from the Global Burden of Disease Study 2021. Front. Aging Neurosci. 16:1498756. 10.3389/fnagi.2024.1498756 39868382 PMC11757241

[B5] Poewe W. Seppi K. Tanner C. M. Halliday G. M. Brundin P. Volkmann J.. (2017). Parkinson disease. Nat. Rev. Dis. Primers 3:17013. 10.1038/nrdp.2017.13 28332488

[B6] Schapira A. H. V. Chaudhuri K. R. Jenner P. (2017). Non-motor features of Parkinson disease. Nat. Rev. Neurosci. 18, 435–450. 10.1038/nrn.2017.62 28592904

